# Spontaneous common bile duct perforation in full term pregnancy: a rare case report and review of literature

**DOI:** 10.1186/s12893-021-01230-2

**Published:** 2021-05-08

**Authors:** Matiullah Masroor, Mohammad Arif Sarwari

**Affiliations:** 1 Department of General Surgery, Amiri Medical Complex, Qargha Road, Afshar, Kabul, Afghanistan; 2 Department of Cardiovascular Surgery, The Second Xiangya Hospital of Central South University, 139 Renmin Middle Road, Changsha, 410011 China

**Keywords:** Spontaneous biliary perforation, Pregnancy, Common bile duct, Peritonitis, Cholecystectomy

## Abstract

**Background:**

Spontaneous biliary system perforation is a rare presentation in clinical practice especially in adults. It is rarely suspected and diagnosed preoperatively due to small number of cases, vague sign and symptoms, and ambiguous presentation.

**Case presentation:**

We describe an interesting case of spontaneous perforation of the common bile duct in a 16 year-old female who presented a week after her first birth to the emergency department with complaints of diffuse abdominal pain, abdominal distention, fever, vomiting, and constipation. She was having generalized peritonitis but the etiology was unclear despite a thorough workup. She underwent exploratory laparotomy, and a perforation in the supra duodenal region of the common bile duct was found intraoperatively. The common bile duct was repaired over T-tube, and cholecystectomy was performed; the patient was recovered uneventfully.

**Conclusion:**

Spontaneous biliary perforation is a rare cause of acute abdomen in adults and extremely rare in pregnancy. Its delayed diagnoses and management can lead to a high morbidity and mortality. All physicians, especially surgeons, should be aware of this possibility and consider it a cause of peritonitis on differential diagnosis particularly when there is no apparent etiology available for presentation.

## Background

Most injuries to the bile duct are iatrogenic after some kind of interventions like endoscopic retrograde cholangiopancreatography (ERCP) as well as open and laparoscopic cholecystectomy [1]. Spontaneous bile duct perforation can also occur, but it is rare in adults. Etiologies include increased pressure in the bile duct secondary to obstruction by stones, strictures, tumors, and parasites; intra mural infection; necrosis of the bile duct wall secondary to thrombosis of intramural vessels; direct erosion by stones; cirrhosis; and weakness of the duct wall for multiple reasons. It can also occur without a known cause, but calculi were found in common bile duct (CBD) in 70% of cases. It is comparatively common in infants and etiologies are congenital biliary anomalies most of the time in this group [2, 3]. Here, we present a young female who was diagnosed with spontaneous CBD perforation during surgery one week after giving birth. We also performed an extensive literature review and found only five cases of bile duct perforation during pregnancy or in the early postpartum period reported in the literature to date.

## Case presentation

A 16 year-old female Gravida 1 para 1 presented to the emergency department with no past medical and surgical history. According to her history, she developed intermittent fever and chills, right upper quadrant pain, and urinary retention for the last 2 weeks; she underwent normal vaginal delivery one week ago. She was hospitalized in another province for these complaints. The presenting complaints were diffuse abdominal pain, abdominal distention, and continuous fever from the last three days with vomiting and constipation for the last two days. The vital signs were BP 133/97 mmHg, RR 20/m, PR 136/m, temperature 39 C^0^, and SpO_2_ 90%. On physical examination, the patient looked ill with a distended abdomen, generalized tenderness, rebound tenderness, and guarding. Fluid thrill and shifting dullness was positive. Bowel sounds were not audible on auscultation. Blood workup showed TLC 21,000/mm^3^ (normal 4000–11,000), neutrophil 89% (normal 40–75), Hb 8.2 g/dl (normal 11.5–16.5), serum creatinine 0.86 mg/dl (normal 0.6–1.2), total bilirubin 0.71 mg/dl (normal 0.1–1.2), SGOT 20 IU/ml (normal 0–40), SGPT 34 U/L (normal 5–40), alkaline phosphatase 109 IU/L (normal 40–240), and blood group AB + ; the HbsAg, anti HCV Ab, and HIV were negative. She was initially received by the emergency doctor and was diagnosed with “postpartum sepsis” because she was having signs and symptoms of sepsis. Ultrasound showed distended bowl loops floating in free fluid in the peritoneal cavity. Erect chest X-ray showed no air under the right hemidiaphragm. CT with contrast was performed revealing gross ascites suggesting peritonitis but the cause of the ascites was unclear.

**Fig. 1:**
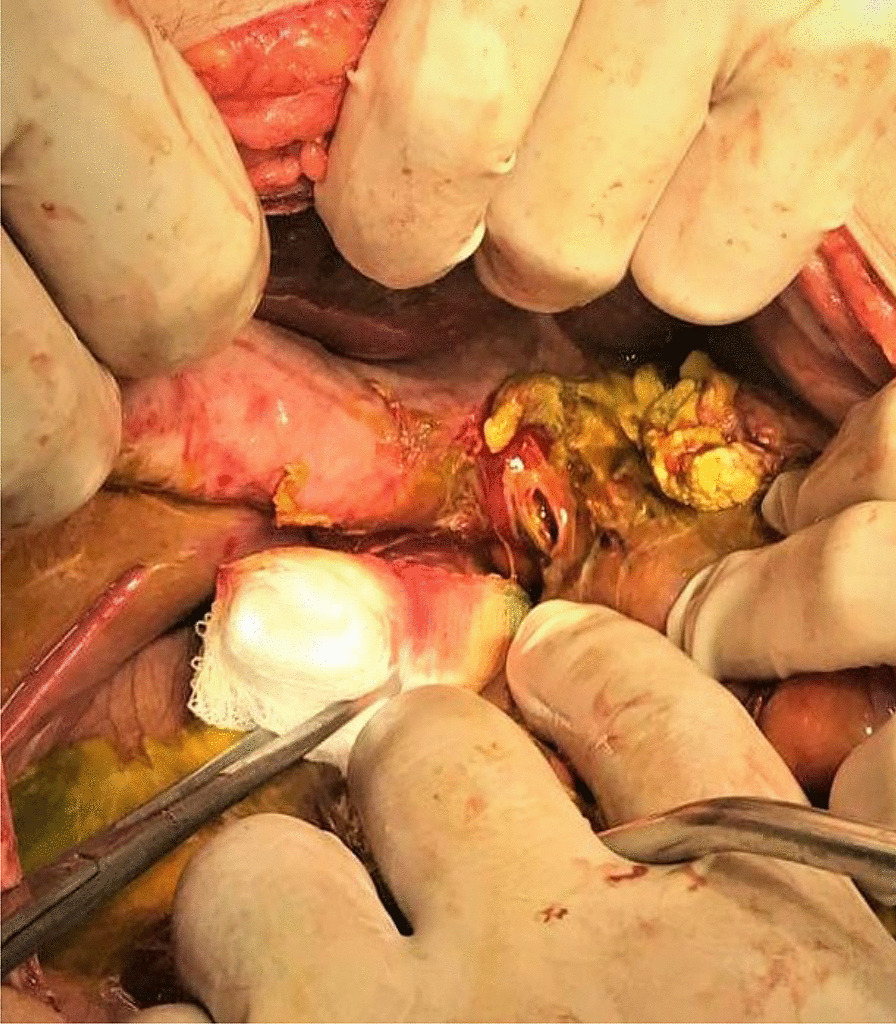
Perforation of supra duodenal segment of the common bile duct

An exploratory laparotomy was performed after initial management with a hollow viscous perforation in mind. Biliary peritonitis was found with about 4 L of bile-stained fluid in the peritoneal cavity; the stomach, small intestine (distended), and colon were normal. A perforation of about 7–8 mm was found on anterolateral wall of supra duodenal segment of CBD below the junction of cystic duct and common hepatic duct as shown in Fig. 1. Gall bladder and the rest of biliary tree was normal without any calculus. An intra-operative cholangiogram was not available so a 10 French feeding tube was passed to the duodenum through a perforation to confirm the distal obstruction that passed without any resistance. Peritoneal lavage with warm normal saline, cholecystectomy, and CBD repair over a T-tube was thus performed. The post-operative stay was uneventful, and the patient fully recovered and was discharged on the 7th post-operative day. The T- tube was removed on the 15th post-operative day after the cholangiogram showed no filling defects and normal contrast flow to the duodenum. The patient did well after one year of follow up.

## Discussion and conclusion

Spontaneous perforation of the common bile duct was first described by John Freeland in 1882 when he found multiple diverticula around CBD with stones on autopsy [4]. Most common sites of biliary system perforation are the gallbladder that account for 91% of cases followed by common bile duct (4.4%), cystic duct (3.3%), and common hepatic duct (1.1%) [5]. Apart from the gallbladder, the most common site of extrahepatic biliary system perforation is the junction of cystic duct with hepatic duct [6]. The first case in infants was presented by Dijkstra in 1932 [7]. The incidence of spontaneous biliary perforation is 1.5 in 1,000,000 live births over the first year of life. The most common site of perforation in infants below age one was the junction of common hepatic and cystic duct (43%), common bile duct (23%), gall bladder (12%), hepatic duct (9%), and cystic duct (5%) [8]. The incidence of overall biliary system disease in pregnancy ranges from 0.05–0.3% [9].

The first case of spontaneous bile duct perforation in pregnancy associated with gall stones was reported by Piotrowski et al*.* in 1990 but bile duct perforation due to congenital anomalies in pregnancy has been reported prior to that in the literature. About 70% of spontaneous biliary perforation cases were associated with gall stones where the stone were found during surgery [2]. The prevalence of spontaneous bile duct perforation is rare in pregnancy with only a few cases reported in the literature. All cases (to the best of our knowledge) of normal anatomy biliary system perforation (including perforation of gallbladder and bile duct) in pregnancy or early postpartum period reported in literature are listed in Table 1. [2, 10–17].

**Table 1:** All reported cases of spontaneous biliary system perforation in pregnancy or early postpartum period

Name and year of publication	Age/GP	No. of cases		Site of perforation	Cause of perforation	Surgery time	Treatment	References
Chaimoff et al. 1973	?	1	Gall bladder		?	?	?	[10]
Piotrowski et al. 1990	23 G1P0	1	CHD		Gall stone	Concomitant with C section	Hepaticojejunostomy	[2]
Behera et al. 1991	?	1	Gall bladder		Acute cholecystitis	?	?	[11]
Goodlin et al. 1991	?	4	Gall bladder		Gangrene of Gall bladder	Postpartum	Cholecystectomy	[12]
Petrozza et al. 1995	28 G7P6 23 G4P4	2	Gall bladder		Gall stone	Postpartum	Cholecystectomy	[13]
McGrath et al. 2005	34 G1P0	1	CBD		Gall stone	Postpartum	Sphincterotomy + Stent placement	[14]
Talwar et al. 2006	28 G2P1	1	Gall bladder		Gall stone	During pregnancy	Cholecystectomy	[15]
Talwar et al. 2006	21 G1P0	1	CBD		Idiopathic	During pregnancy	Over T-tube repair	[15]
Dabbas et al. 2008	20 G1P1	1	CBD		Gall stone	Postpartum	Choledocholithotomy + Over T-Tube repair	[16]
Bediako-Bowan et al. 2013	29 ?	1	CBD		Idiopathic	Postpartum	Cholecystectomy + Over T-Tube repair	[17]
Masroor et al. 2021	16 G1P1	1	CBD		Idiopathic	Postpartum	Cholecystectomy + Over T-Tube repair	Current study

*CHD* Common hepatic duct, *CBD* Common bile duct, *GP* Gravida and Para

Most cases are not spontaneous because they are secondary to some other underlying pathology. Out of all these spontaneous biliary system perforations cases given in the Table 1, only five cases of bile duct perforation have been reported: four in the common bile duct and one in the common hepatic duct. We present here a truly spontaneous common bile duct perforation case that we think is the 6th case of the bile duct and 5th case of CBD perforation during pregnancy in the literature.

According to our analysis, the most common site of biliary tract perforation during pregnancy is gall bladder (9/15; 60%), CBD (5/15; 33.3%), and hepatic duct (1/15; 6.66%). The causes of spontaneous perforation include gall stones (6/9; 66.6%) and idiopathic (3/9; 33.3%). The causes of the six other cases mentioned in the Table 1 have not been clearly described, and they have been excluded from the analysis for the purpose of accuracy.

The theories behind spontaneous bile duct perforation in adults are obstruction distal to perforation leading to high canalicular pressure, weakness of bile duct wall, or a combination of both. The etiologies leading to these phenomena mentioned in the literature are impacted stones or erosion of bile duct wall by stones without impaction, intramural infection, strictures, tumors, parasites, spasm of sphincter of Oddi, necrosis of the wall of the bile duct due to thrombosis of bile duct blood vessels, cirrhosis, birth trauma, biliary tract congenital anomalies like choledochal cyst or biliary diverticulum, connective tissue diseases, and previous biliary tract surgeries. Other comorbidities associated with spontaneous biliary perforation include HIV infection, tuberculosis of CBD, Hodgkin’s Lymphoma, severe necrotizing enterocolitis involving duodenum, and viral infection of the bile duct [2, 3, 6]. Spontaneous bile duct perforation sometimes can be idiopathic like in our case but the possible reason for idiopathic cases might be thrombosis of those small blood vessels leading to ischemia and necrosis of bile duct wall and finally perforation.

The presentation of the patient can be different from case to case because it can have both acute and insidious onset. Most patients having insidious onset (80%) may present with abdominal distention without abdominal pain and clay color stool; progressive jaundice may follow [3, 6, 18]. In acute cases (20%), the signs and symptoms of acute abdomen like generalized abdominal pain, abdominal distention due to bilious ascites, vomiting, fever, jaundice, high levels of bilirubin, or even shock may occur. The patient can present with a perihepatic collection or abscess instead of generalize peritonitis if the bile is localized to the area [3, 19].

We believe our case was insidious onset and developed CBD perforation two weeks before she was presented to us but she was misdiagnosed by less experienced local health care workers in the rural area perhaps because she was a full term pregnant and they misinterpreted the symptoms as labor pain. She gave birth to her baby just one week after her symptoms started. She finally developed generalized abdominal pain that might be due to the rupture of the walled off biloma leading to fulminant peritonitis, infection with high fever, and high leukocyte counts or symptoms that became obvious when enough bile accumulated and infected within the peritoneal cavity. There is less of a chance of the first scenario because we could not find a walled-up cavity during surgery. The symptoms of bowel obstruction (abdominal pain, distention, vomiting, and constipation) can be explained by paralytic ileus due to infection and peritonitis.

Such cases are rare, and suspicion and preoperative diagnosis of the condition is difficult. Diagnosis is mostly made during surgery. Perforation of the biliary system is a known complication of cholelithiasis or choledocholithiasis, and one should suspect biliary perforation if the patient presents with perihepatic abscess or signs and symptoms of peritonitis with a history of biliary stone disease [19]. If suspected, pre-operative diagnosis can be made by hepatobiliary iminodiacetic acid scan (HIDA scan), magnetic resonance cholangiopancreatography (MRCP), endoscopic retrograde cholangiopancreatography (ERCP), magnetic resonance imaging (MRI), and computerized tomography (CT) scan. These facilities are expensive and not widely available especially in low-income countries or in rural areas of many other countries; thus, an easy and cheaper biochemical test from the ascitic tap “ascitic fluid bilirubin concentration, and ascitic fluid to serum bilirubin ratio” can help to diagnose preoperative biliary peritonitis. The normal range of ascitic fluid bilirubin is 0.7–0.8 mg/dl, and concentrations above 6 mg/dl support the diagnosis of choleperitoneum [18]. A study by Darwin et al. showed that a peritoneal fluid to serum bilirubin ratio (FSBR) greater than 5 is 100% specific and sensitive for prediction of bile leak [20]. Ultrasonography is a first study because it is cheap, readily available, can show free fluid in the abdominal cavity, fluid and pus collection in perihepatic area, and biliary system pathologies. In the case of CBD stones, the dilated biliary tree proximal to the obstruction can be nicely visualized as well; however, ultrasound may not find the exact site of the perforation but it is still a helpful investigation especially in low-resource settings. Chest X-rays in erect or left lateral decubitus positions will not reveal gas under the right hemidiaphragm; this is expected in many gut perforations cases. Thus, the suspicion should be higher if the patient is presented with peritonitis but with no gas under right hemidiaphragm on X ray, and the presence of other positive markers like bilious peritoneal tap with a history of biliary disease. Sharma et al. recommended peritonitis with bilious peritoneal tap, no pneumoperitoneum, and acholic stool pathognomonic for spontaneous biliary perforation [21].

The condition may be confused and challenging to diagnose because of similar presentation with other pregnancy-related diseases like pregnancy induced hypertension (PIH), preeclampsia, and hemolysis, elevated liver enzymes, and low platelet count syndrome (HELLP). McGrath et al. and Goodlin et al. discussed biliary system perforation confused with or misdiagnosed as pregnancy-associated problems [12, 14]. This confusion may lead to delayed diagnosis that can lead to high morbidity and mortality. Spontaneous perforation of the biliary tree might be considered in differential diagnosis. Multidisciplinary approaches need to be adopted in such cases to avoid catastrophic events like these.

Management of spontaneous bile duct perforation ranges from minimally invasive intervention to more aggressive surgical intervention. Based on the patient’s condition, different treatment modalities can be chosen. The management mainstay in the case of suspected calculi in the biliary tree is threefold: 1) evaluate stone probability in biliary tree, 2) choledocholithotomy or removal of stones if present, and 3) removal of the source of stone (cholecystectomy) [22]. ERCP is of diagnostic and therapeutic advantage, and spontaneous biliary perforation cases can be diagnosed and treated at the same time with removal of the stone and placement of stent in the bile duct [14, 21]. During failed ERCP cases or where ERCP facilities is not available, one can use laparoscopic or open exploration. If there is biloma and localized perihepatic abscess secondary to CBD obstruction by stone, then it can be drained percutaneously concomitantly with stones in the CBD removed via endoscopic sphincterotomy [19].

Patients presented with generalized biliary peritonitis need prompt exploration and thorough peritoneal drainage followed by management of the perforation site [21]. An intraoperative cholangiogram must be performed if available to check for stones and pathologies of the biliary tree. A conservative management of abdominal drainage and biliary tree decompression is recommended if there is no post-perforation obstruction; perforation will heal automatically once the biliary tree is decompressed [19]. Primary closure of the perforation site is hard and dangerous to perform because of inflammation around the perforation site; most patients will recover after external decompression and after treating the primary pathology [19, 21].

Some authors believe that primary closure of the bile duct perforation can be performed if the facility of intraoperative cholangiogram is available and there is no distal obstruction; however, a biliary enteric anastomosis is required to avoid portal hypertension and biliary cirrhosis if there is distal obstruction like stricture or atresia [3, 23]. Spigland et al. also suggested only external drainage for biliary perforation without ductal abnormalities but added that cholecystostomy may help in healing of duct perforation and also guide when to remove peritoneal drain [24]. Gurusamy et al. published a review study in 2007 and suggested that primary closure of choledochotomy is as effective as closure over T-tube in open exploration of the bile duct; another review published by the same author in 2013 suggested that choledochotomy closure over T-tube in open exploration of bile duct increases operative time and hospital stay without any benefit. They believed that routine T-tube drainage for CBD stones should be avoided because there is no justification for its use based on current available evidence. They also suggested that T-tube drainage in laparoscopic exploration of CBD also increases surgery time and post-operative hospital stay without significant difference in morbidity than primary closure without stent [25]. Simple T-tube peritoneal drainage is also acceptable even in the presence of distal obstruction when the exploration of porta hepatis is very risky in inflamed conditions, which may worsen the scenario. This mini surgery is less morbid and has good chances of healing. At the minimum, it will stabilize the patient for second surgery if the condition has not cured [23]. We recommend the use of peritoneal drainage and percutaneous cholecystostomy in cases where porta hepatis exploration is not feasible for any reason even if distal obstruction is present. Roux-en-Y bilioenterostomy was performed previously for biliary perforation but is now limited to untreatable distal obstruction, persistent biliocutaneous fistulae, or biliary leakage and CBD perforation associated with choledochal cysts [21]. If cholangiogram is not available, then the patient may be best managed by closure over the T- tube and cholecystectomy as in our case [3, 18, 23].

Spontaneous bile duct perforation is very rare in adults and even rarer during pregnancy. A preoperative diagnosis is hard to make because of its rarity and similar presentation with many other diseases. Physicians (especially surgeons) should be aware of this condition and must consider it a cause of peritonitis on differential diagnosis. It is best to strongly suspect patients presented with peritonitis in pregnancy or in early post-partum period with bilious peritoneal tap, without pneumoperitoneum, and no apparent cause for peritonitis. Peritoneal tap bilirubin concentration and its ratio with serum bilirubin are very helpful in diagnosis. The purpose of the management is to halt the catastrophic event, treat, and then remove the cause and source of perforation whenever possible. One should ultimately decompress the biliary tree. Simple peritoneal drainage can be done in patients unfit for porta hepatis exploration followed by another surgery after stabilization.

## Data Availability

The datasets used during the current study are available from the corresponding author on reasonable request.

## Abbreviations

CBD: Common bile duct
HIDA: Hepatobiliary iminodiacetic acid
MRCP: Magnetic resonance cholangiopancreatography
ERCP: Endoscopic retrograde cholangiopancreatography
MRI: Magnetic resonance imaging
CT: Computerized tomography
FSBR: Fluid to serum bilirubin ratio
PIH: Pregnancy induced hypertension
HELLP: Hemolysis, elevated liver enzymes, Low platelets
